# Nrf2 Inhibits Periodontal Ligament Stem Cell Apoptosis under Excessive Oxidative Stress

**DOI:** 10.3390/ijms18051076

**Published:** 2017-05-17

**Authors:** Yanli Liu, Hongxu Yang, Yi Wen, Bingyi Li, Yinhua Zhao, Jing Xing, Min Zhang, Yongjin Chen

**Affiliations:** 1 State Key Laboratory of Military Stomatology and National Clinical Research Center for Oral Diseases and Shaanxi International Joint Research Center for Oral Diseases, Department of General Dentistry and Emergency, School of Stomatology, the Fourth Military Medical University, 145 Changle West Road, Xi’an 710032, China; fmmulh@fmmu.edu.cn (Y.L.); 18082208000@163.com (Y.Z.); 2 State Key Laboratory of Military Stomatology and National Clinical Research Center for Oral Diseases and Shaanxi International Joint Research Center for Oral Diseases, Department of Oral Anatomy and Physiology and TMD, School of Stomatology, the Fourth Military Medical University, 145 Changle West Road, Xi’an 710032, China; yanghongxushmily@163.com; 3 State Key Laboratory of Military Stomatology and National Clinical Research Center for Oral Diseases and Shaanxi Clinical Research Center for Oral Diseases, Department of Orthodontics, School of Stomatology, the Fourth Military Medical University, 145 Changle West Road, Xi’an 710032, China; wy162729@126.com; 4 The cadet brigade, the Fourth Military Medical University, 169 Changle West Road, Xi’an 710032, China; lby962396076@163.com (B.L.); xingjing123@163.com (J.X.)

**Keywords:** periodontitis, periodontal ligament stem cells, nuclear factor-erythroid 2-related factor 2, oxidative stress, apoptosis

## Abstract

The present study aimed to analyze novel mechanisms underlying Nrf2-mediated anti-apoptosis in periodontal ligament stem cells (PDLSCs) in the periodontitis oxidative microenvironment. We created an oxidative stress model with H_2_O_2_-treated PDLSCs. We used real-time PCR, Western blotting, TUNEL staining, fluorogenic assay and transfer genetics to confirm the degree of oxidative stress and apoptosis as well as the function of nuclear factor-erythroid 2-related factor 2 (Nrf2). We demonstrated that with upregulated levels of reactive oxygen species (ROS) and malondialdehyde (MDA), the effect of oxidative stress was obvious under H_2_O_2_ treatment. Oxidative molecules were altered after the H_2_O_2_ exposure, whereby the signaling of Nrf2 was activated with an increase in its downstream effectors, heme oxygenase-1 (HO-1), NAD(P)H:quinone oxidoreductase 1 (NQO1) and γ-glutamyl cysteine synthetase (γ-GCS). Additionally, the apoptosis levels gradually increased with oxidative stress by the upregulation of caspase-9, caspase-3, Bax and c-Fos levels in addition to the downregulation of Bcl-2. However, there was no alterations in levels of caspase-8. The enhanced antioxidant effect could not mitigate the occurrence of apoptosis. Furthermore, Nrf2 overexpression effectively improved the anti-oxidative levels and increased cell proliferation. At the same time, overexpression effectively restrained TUNEL staining and decreased the molecular levels of caspase-9, caspase-3, Bax and c-Fos, but not that of caspase-8. In contrast, silencing the expression of Nrf2 levels had the opposite effect. Collectively, Nrf2 alleviates PDLSCs via its effects on regulating oxidative stress and anti-intrinsic apoptosis by the activation of oxidative enzymes.

## 1. Introduction

Periodontitis is highly prevalent and can affect up to 90% of the worldwide population. Furthermore, it is also known as the leading killer in oral health [1]. Periodontitis is a general term that is used to describe specific diseases that damage the gingiva and the supporting connective tissue, as well as the alveolar bone, which anchor the teeth in the jaws [2]. In recent years, stem cell technology and the development of tissue engineering technology have brought new opportunities for repairing periodontal tissue defects. Therefore, some scholars, through animal experiments, have confirmed that using mesenchymal stem cells (MSCs) with exogenous bone graft composite material for repairing periodontal tissue defects is better than single bone graft material repairs [3]. Periodontal ligament stem cells (PDLSCs) are a type of MSCs. Many studies have found that using PDLSCs to repair non-inflammatory alveolar bone defects works better than repairing periodontitis bone defects [4].

Studies in recent years affirmed that periodontal local tissue generates interleukine-8 (IL-8) and macrophage colony stimulating factor from neutral polymorphonuclear leukocytes (PMNs), when inflammatory lesions occur in the periodontitis [5,6]. PMNs act as a primary defense against outside microbial invasions and after swallowing pathogens, their oxidases are activated. Therefore, reactive oxygen species (ROS), such as hydrogen peroxide (H_2_O_2_), produced by PMNs were found around the periodontal tissue in the periodontitis microenvironment [7].

Oxidative stress (OS), which is mediated by ROS, such as H_2_O_2_, plays a crucial role in the periodontitis [8]. Sub-lethal concentrations of H_2_O_2_ can damage the periodontal tissue and could influence the efficiency of repair of periodontal tissue defects by PDLSCs in periodontitis [9]. Studies demonstrated that a minimum of 50 µM H_2_O_2_ could induce OS [10]. OS activated by periodontal microenvironment directly or indirectly, could damage PDLSC proteins, lipids, nucleic acids and other macromolecular substances that have physiological functions, which leads to low metabolic activity and cell cycle arrest by activating apoptosis [11,12]. Apoptosis is a type of programmed cell death that is used to eliminate excessively damaged or stressed cells throughout life in a variety of organisms, thus maintaining normal development, tissue remodeling and homeostasis [13]. Over the past decade, apoptosis has been identified as a critical factor responsible for the damage and differentiation of PDLSCs, with excessive OS potentially being able to destroy their DNA through cell apoptosis [14]. OS is one of the most important factors that influences stem cells. It can affect the differentiation of PDLSCs and even cause cell death to reduce the effectiveness of defect repair [15].

It is well known that the nuclear factor-erythroid 2-related factor 2 (Nrf2) signaling pathway has an antioxidant effect and is involved in the cellular antioxidant defense system [16,17]. Under normal conditions, Nrf2 exists in the cell, combined with Kelch-like ECH-associated protein 1 (Keap1). In response to OS, the cysteine residues of Keap1 are modified, which causes a conformational change that leads to the release of Nrf2 and its phosphorylation by the protein kinase C (PKC). Additionally, this reduces the protein kinase recognition, increases the content and stability of Nrf2 in addition to promoting its translocation into the nucleus. Nrf2 upregulates the expression of antioxidants and detoxifying genes by binding to antioxidant response elements (AREs) in the promoter region of the encoding genes, which starts the transcription of downstream target genes [18]. Research has confirmed that the phosphorylation of Nrf2 plays a main mechanistic role in oxidative stress resistance [19]. On one hand, Nrf2/ARE activation may inhibit pro-inflammation, including cytokines, inflammatory chemokines, cell adhesion factors, matrix metalloproteinases and inducible nitric oxide synthase (iNOS) [20,21]. On the other hand, it can increase a variety of downstream phase II detoxifying enzymes and antioxidant gene expression, including Heme oxygenase-1 (HO-1), catalase, superoxide dismutase (SOD), gamma-glutamyl cysteine synthetase (γ-GCS) and NAD(P)H:quinone oxidoreductase 1 (NQO1) [22,23,24,25].

To date, the role of Nrf2 in the occurrence and development of periodontitis is still unclear. Considering the above problems and combining the research from previous experimental results, we have formulated the following hypothesis: the local oxidative stress microenvironment in periodontitis can lead to the oxidation imbalance of PDLSCs, which can accelerate their apoptosis and influence their repair regeneration function. At the same time, endogenous antioxidant molecules, such as Nrf2, may play an important role in the process of this disease. The purpose of this study was to examine the mechanisms of Nrf2-mediated anti-apoptosis in PDLSCs in the OS microenvironment.

## 2. Results

### 2.1. Culture and Identification of PDLSCs

**Figure 1:**
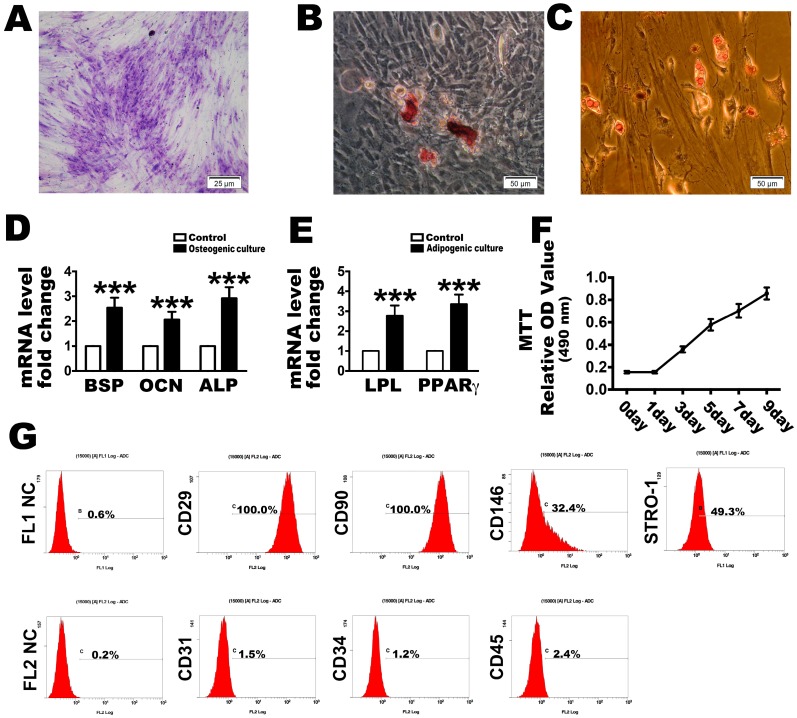
Identification of human periodontal ligament stem cells (PDLSCs) used in this research. Representative image of colony-forming units and (**A**) a random single-cell clone on day 14. Bars = 25 µm; (**B**) Representative images of mineralized cell nodules following a 21-day osteogenic induction period and (**C**) lipid droplets following a 14-day adipogenic induction period. Bars = 50 µm; (**D**) Osteogenesis and (**E**) adipogenesis mRNA expression after the culture conditions compared with control; (**F**) Proliferation of locally isolated PDLSCs, as assessed by MTT assays after cultured at days 1, 3, 5, 7 and 9; (**G**) Cell surface markers identified by flow cytometric analysis. FL1 NC and FL2 NC means the negative control to the FL1 and FL2, respectively. The data are expressed as the mean ± SD. *n* = 5. *** *p* < 0.001 represent significant differences between the indicated columns.

After the limiting dilution technique was used to purify stem cells from the primary cells, passages 3 (P3) were used in the subsequent investigations. The PDLSCs were found mostly in their long spindle-like morphology at P3. Through toluidine blue staining or osteogenesis and adipogenesis evaluations, all of the PDLSCs exhibited the ability to form colonies (Figure 1A) or to transdifferentiate into osteogenic and adipogenic lineages in vitro (Figure 1B,C). Quantification real time PCR results indicated that under osteogenic culture conditions for 21 days, PDLSCs expressed higher levels of osteogenesis-related genes such as *BSP*, *OCN* and *ALP* (Figure 1D) than cells grown in the control medium. At the same time, the adipogenesis-related genes *LPL* and *PPARγ* were expressed at higher levels in the adipogenic-inducible condition than in the control medium after 14 days (Figure 1E). These P3 cells also had a strong proliferation ability, as demonstrated with an MTT assay after cultured at days 1, 3, 5, 7 and 9 (Figure 1F). Similarly, they were positive for the MSC surface markers, CD29, CD90, CD146 and STRO-1, but negative for the hematopoietic and endothelial surface markers, CD31, CD34 and CD45 (Figure 1G).

### 2.2. H_2_O_2_ Stimulation Leads to OS and Nrf2 Signaling Changes

**Figure 2:**
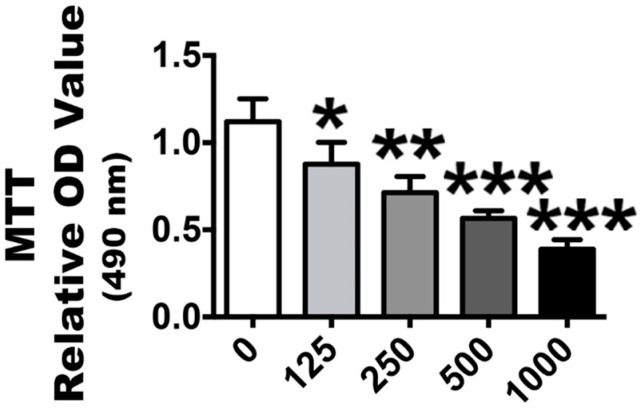
Proliferation of passage 3 PDLSCs after H_2_O_2_ stimulation. The ability of cells proliferation was assessed by MTT assays. The columns represent the mean values with SD, *n* = 5. Different H_2_O_2_ concentration treatments containing 125, 250, 500 and 1000 µmol/L were compared with a 0 µmol/L control group. * *p* < 0.05, ** *p* < 0.01 and *** *p* < 0.001 represent significant differences between the indicated columns.

**Figure 3:**
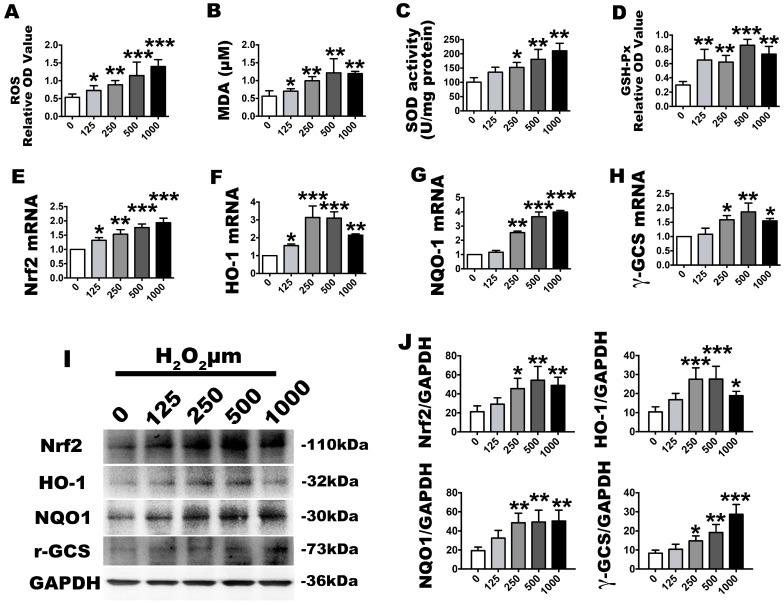
Effects of the H_2_O_2_ treatment model, as described in the group design, on cell oxidative stress and anti-oxidative changes. The corresponding ROS level were assessed by (**A**) absorbance OD values, (**B**) MDA expression levels and (**C**) superoxide dismutase (SOD) and (**D**) GSH-Px absorbance OD values; (**E**–**H**) Relative *Nrf2*, *HO-1*, *NQO1* and *γ-GCS* molecular gene expression levels, as determined by real-time PCR analysis; (**I**) Western blot images for nuclear factor-erythroid 2-related factor 2 (Nrf2), heme oxygenase-1 (HO-1), NAD(P)H:quinone oxidoreductase 1 (NQO1) and γ-glutamyl cysteine synthetase (γ-GCS) and (**J**) their quantification. Data are presented as the means ± SD, *n* = 5. Different H_2_O_2_ concentration treatments containing 125, 250, 500 and 1000 µmol/L were compared with a 0 µmol/L control group. * *p* < 0.05, ** *p* < 0.01 and *** *p* < 0.001 represent significant differences between the indicated columns.

In vitro, we stimulated human P3 PDLSCs with a gradient of H_2_O_2_ concentrations to induce OS. After exposure to 125, 250, 500 or 1000 µmol/L of H_2_O_2_ for 2 h, the effect of OS model with different concentrations was investigated with an MTT assay, which revealed that cytotoxicity positively correlated with low to high H_2_O_2_ concentrations (Figure 2). The 1000 µmol/L concentration was the most cytotoxic to the cells. After it was established that OS was affecting the PDLSCs, the OS molecular markers, ROS, which have important roles in cell signaling and damage, were analyzed with a fluorogenic assay. This showed that the ROS activation level was significantly elevated at all stimulation concentrations with increasing OS levels (Figure 3A). Similar results were found in the expression of malondialdehyde (MDA) (Figure 3B), which occurs naturally and is a marker for lipid peroxidation. However, regarding the phase II detoxifying enzymes, the SOD and GSH-Px levels were also increased (Figure 3C,D). Additionally, the basal Nrf2 mRNA and protein expression levels were significantly upregulated (Figure 3E,I,J) (Nrf2 positive and negative control included in Supplemental Figure S1A,B). The mRNA and protein levels of HO-1, NQO1 and γ-GCS, which are downstream of Nrf2, were significantly increased with increasing exposure concentrations (Figure 3F–J). These results indicate that the OS reaction was obvious because the oxidative and anti-oxidative molecules increased after H_2_O_2_ exposure, while the anti-oxidative signaling of Nrf2 was also activated.

### 2.3. OS Induces PDLSC Apoptosis

**Figure 4:**
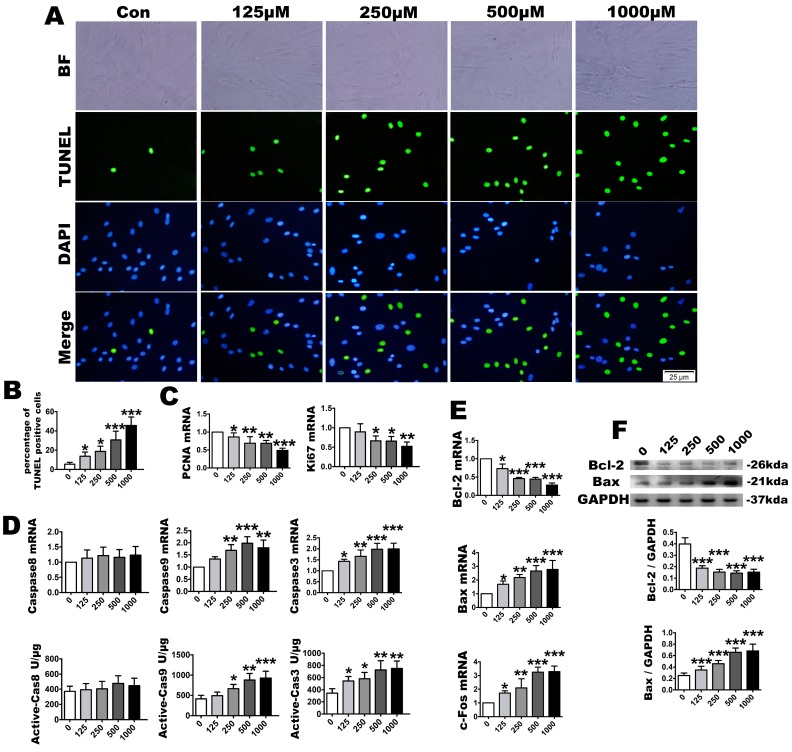
After H_2_O_2_ treatment, identification of cell proliferation and apoptosis. Representative cell apoptosis images, as assayed by (**A**) TUNEL staining and (**B**) its quantification; The TUNEL staining were accompanied with a nuclear stain and morphological by DAPI and BF. BF means bright fields. DAPI means 4′,6-diamidino-2-phenylindole. Bars = 25 µm; (**C**) Relative proliferation parameters of PCNA and Ki67, as determined by real-time PCR analysis; (**D**) Caspase-8, caspase-9 and caspase-3 mRNA expression and active unit levels; Relative Bcl-2, Bax and c-Fos expression levels, as assayed by (**E**) real-time PCR and (**F**) Western blotting. Data are presented as the means ± SD, *n* = 5. Different H_2_O_2_ concentration treatments containing 125, 250, 500 and 1000 µmol/L compared with a 0 µmol/L control group. * *p* < 0.05, ** *p* < 0.01 and *** *p* < 0.001 represent significant differences between the indicated columns.

As sub-lethal H_2_O_2_ concentrations can directly or indirectly produce damage to nucleic acids and cellular proteins, we next evaluated the extent of apoptosis by TUNEL staining, fluorogenic assay, RT-PCR and Western blotting to identify the effect of the OS microenvironment on PDLSCs. TUNEL staining indicated that with the augmentation of oxide stimulation in the microenvironment, the TUNEL-positive cells were significantly increased compared with the control group. The highest positive rate was observed in the 1000 µmol/L group (Figure 4A,B). The PCNA and Ki67 mRNA levels, a main proliferation indicator, were significantly downregulated in the 125 to 1000 µmol/L concentrations (Figure 4C). The caspase-3 and caspase-9 mRNA expression levels were upregulated at all concentrations. However, no upregulation was detected in the mRNA expression level of caspase-8. Furthermore, similar to the gene expression levels, the fluorogenic analysis indicated that the active caspase-9 and caspase-3 levels were increased following exposure, although the the level of active caspase-8 did not increase (Figure 4D). The mRNA levels of the anti-apoptosis molecule, Bcl-2, was downregulated. In contrast, the mRNA levels of the apoptosis-promoting molecules, Bax and c-fos, were upregulated. The Bcl-2 and Bax Western blot results were consistent with the mRNA level changes (Figure 4E,F). These findings suggest that the apoptosis levels increased gradually with the augmented oxidative stress and that the enhanced antioxidant effect could not protect against the apoptosis.

### 2.4. Nrf2 Is a Key Molecule in PDLSC Apoptosis Protection during OS

**Figure 5:**
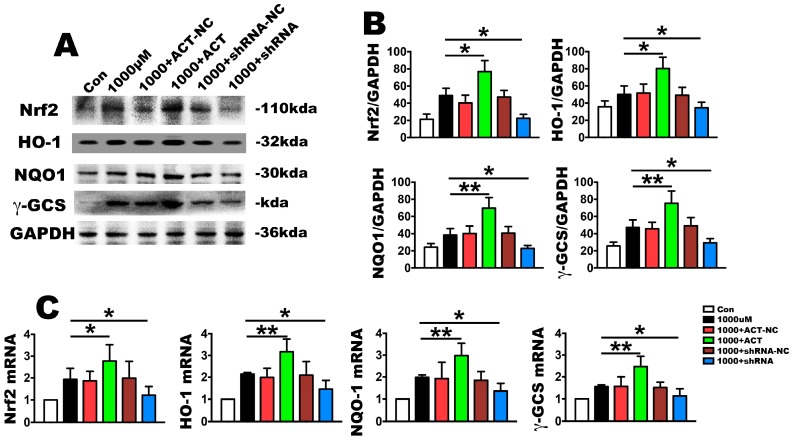
Nrf2 influences OS mediated gene expression in PDLSC. The PDLSCs were treated with 1000 µmol/L H_2_O_2_ for 2 h. The PDLSCs were transfected with lentiviral Nrf2 for overexpression (1000 + ACT: overexpression of Nrf2 at 1000 µmol/L H_2_O_2_ for 2 h) or a lentiviral control (1000 + ACT-NC: overexpression negative control at 1000 µmol/L H_2_O_2_ for 2 h), and they were silenced with Nrf2 shRNA (1000 + shRNA: silence of Nrf2 at 1000 µmol/L H_2_O_2_ for 2 h) or control shRNA (1000 + shRNA-NC: silence negative control at 1000 µmol/L H_2_O_2_ for 2 h). (**A**) Relative gene expression levels of Nrf2 and its related downstream molecules, HO-1, NQO1 and γ-GCS, as determined by real-time PCR analysis; (**B**) Western blot for the anti-oxidative molecules, Nrf2, HO-1, NQO1 and γ-GCS and (**C**) their quantification. Data are presented as the means ± SD, *n* = 5. * *p* < 0.05 and ** *p* < 0.01 represent significant differences between the indicated columns.

**Figure 6:**
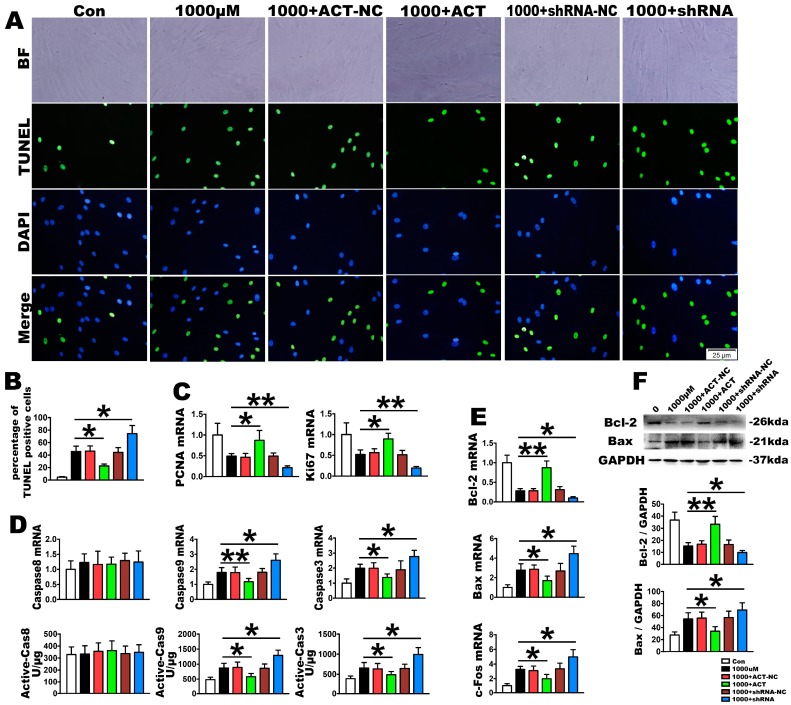
OS-induced PDLSC apoptosis changes through Nrf2 overexpression or silencing. PDLSCs were treated with 1000 µmol/L H_2_O_2_ for 2 h. The PDLSCs were transfected with lentiviral Nrf2 for overexpression (1000 + ACT: overexpression of Nrf2 at 1000 µmol/L H_2_O_2_ for 2 h) or a lentiviral control (1000 + ACT-NC: overexpression negative control at 1000 µmol/L H_2_O_2_ for 2 h), and they were silenced with Nrf2 shRNA (1000 + shRNA: silence of Nrf2 at 1000 µmol/L H_2_O_2_ for 2 h) or control shRNA (1000 + shRNA-NC: silence negative control at 1000 µmol/L H_2_O_2_ for 2 h). Representative cell apoptosis images, as assayed by (**A**) TUNEL staining and (**B**) their quantification; The TUNEL staining were accompanied with a nuclear stain and morphological by DAPI and BF. BF means bright fields. Bars = 25 µm; (**C**) Relative proliferation parameters of PCNA and Ki67, as determined by real-time PCR analysis; (**D**) Caspase-8, caspase-9 and caspase-3 mRNA expression and active unit levels; Expression levels of the relative apoptosis molecules, Bcl-2, Bax and c-Fos, as assayed by (**E**) real-time PCR and (**F**) Western blotting. Data are presented as the means ± SD, *n* = 5. * *p* < 0.05 and ** *p* < 0.01 represent significant differences between the indicated columns.

To investigate the effect of Nrf2 on H_2_O_2_-induced apoptosis signaling cascades, Nrf2 silencing or overexpression through siRNA were used in PDLSCs. After treatment with 1000 µmol/L H_2_O_2_, the mRNA and protein of Nrf2 was upregulated in the overexpression group and downregulated in the silencing group, with no change observed in the negative control (Figure 5A–C). The same trends were also observed because the mRNA and protein levels of NQO1, HO-1and γ-GCS, which are the downstream targets of Nrf2, were significantly consistent with this effect (Figure 5A–C). Furthermore, the upregulated Nrf2 produced a significantly decreased TUNEL-positive cell rate (Figure 6A,B), caspase-9 and caspase-3 mRNA levels as well as active caspase-9 and caspase-3 units, but it had no effect on caspase-8 activation compared with the OS stimulation (Figure 6D). It also increased cell proliferation with the upregulation in the mRNA of PCNA and Ki67 (Figure 6C). The anti-apoptosis ability of Nrf2 was revealed by upregulating the mRNA and protein levels of Bcl-2 in addition to downregulating Bax and c-fos (Figure 6E,F). In contrast, silencing Nrf2 downregulated proliferation, as demonstrated by PCNA and Ki67 mRNA expression, and it increased the TUNEL-positive cells as well as the activity of caspase-9 and caspase-3, but had no influence on caspase-8. Finally, this silencing upregulated the mRNA and protein expression levels of Bax and downregulated Bcl-2 (Figure 6A–F). Overall, these results further corroborate the role of Nrf2 signaling in the improvement in anti-oxidative function of PDLSCs, as demonstrated by cell proliferation and anti-apoptosis enhancement, in H_2_O_2_-induced OS.

## 3. Discussion

Periodontal diseases are highly prevalent and are among the most common chronic disorders. They can affect up to 90% of the population and have plagued humans for centuries [2]. Research studies have indicated that partial oxidation of the microenvironment during periodontitis is an important reason for increased periodontal tissue damage [7]. Currently, doctors recognize that the most important methods of curing periodontitis are sequential pre-treatment measures, such as eliminating local stimuli and plaque control, including foundation treatment, surgical treatment, repair and support treatment [26]. Other regenerative therapies, such as bone grafting, guided tissue regeneration and enamel matrix derivative product application have routinely been used in clinical treatment to induce periodontal tissue regeneration [27,28]. However, the current tissue repair treatment methods are not desirable, which may be because of the lack of seed cells in tissue regeneration [3,29]. PDLSCs are a type of MSCs which have high efficient repair to the tissue defects. Stem cell-based periodontal regeneration is being developed rapidly, and dental stem cells, such as PDLSCs are increasingly being investigated as easily accessible undifferentiated cells [30]. However, the outcomes of these stem cells therapies have been limited because they have failed to maintain the PDLSCs ability of regeneration at the OS microenvironment exists in periodontitis [9]. Also the oxidation could influence the repair effect of PDLSCs regeneration in periodontal local tissue [9]. Previous preliminary studies found that the effect of bone defect repair using PDLSCs for periodontitis is negatively related to the degree of local oxidation in the microenvironment. This demonstrates that the oxidative environment is the main reason for the functional and repair ability of PDLSCs. In the present study, PDLSCs that were isolated from human teeth showed characteristics of MSCs that were positive for CD29, CD90, CD146 and STRO-1 surface markers in addition to being negative for CD31, CD34 and CD45. We demonstrated that stimulation of PDLSCs with high concentrations of H_2_O_2_ led to a strong OS reaction with increased ROS and MDA, which was accompanied by a rise of anti-oxidant enzymes from PDLSCs, such as SOD and GSH-Px. MDA is a natural product formed in all mammalian cells as a product of lipid peroxidation and as a marker for oxidative stress. This suggests that exposure to excessive H_2_O_2_ could imitate the microenvironment, in which PDLSCs are located in periodontitis patients. 

DNA structure is an important target of ROS attack. Oxidative damage can directly lead to the rupture of DNA strands, locus mutation and acceleration of cell apoptosis [13,31]. Apoptosis has two main pathways: the mitochondria and death receptor pathways [32,33]. Fas is a member of the tumor necrosis factor receptor superfamily. It is a transmembrane protein and combined with FasL, it can initiate death receptor apoptosis signal transduction, which plays a key role in the caspase cascade pathways [34]. Fas combines with FasL to form a death domain (DD), which contains a 60–70 amino acid sequence [33]. As its N terminal contains a Fas-associated death domain (FADD) structure in the cytoplasm, which is called the death effect domain, its structure can activate the apoptosis signaling protease, caspase-8. Active caspase-8 can mediate its effects through caspase-3 to induce DNA degradation and cell apoptosis, including DNA breaks and chromosome condensation. Its formation in the nucleosome and concentrations in the cytoplasm complete the Fas-mediated death receptor apoptosis pathway [35,36]. Bax, a pro-apoptotic protein of the Bcl-2 family, is a key regulator of apoptosis. As reported in many apoptotic paradigms, Bax resides primarily in the cytosol of healthy cells and is usually found in an inactive state. In the mitochondria apoptosis pathway, Bax undergoes specific conformational changes as well as activating caspase-9 and cytochrome c, which leads to the activation of effector caspase-3 [37,38]. In our results, caspase-3 activation, which has been shown to be upregulated in TUNEL-positive cells, revealed that OS could induce apoptosis in PDLSCs. Caspase-9, not caspase-8, activation indicated that our H_2_O_2_-induced OS promoted the mitochondria apoptosis pathway and not the death receptor pathway.

Further, the oxidative or electrophilic modification of Keap1 or the phosphorylation of serine 40 on Nrf2 by protein kinase C results in the stabilization and release of Nrf2 from Keap1. Nrf2 is the key factor in the oxidative stress reaction in a cell and is the central regulator of the cell oxidation reaction. In normal conditions, the organism uses a series of antioxidant systems and the expression of phase II detoxifying enzymes to remove excess ROS that contains SOD, GSH-Px, catalase and reduced glutathione (GSH) [39,40]. In the body, the Nrf2/ARE signaling pathway acts as an important endogenous anti-oxidative stress pathway [41]. Under oxidative stress, Nrf2 binds to a DNA promoter and initiates the transcription of anti-oxidative genes that encode and modulate the expression of various antioxidant genes, enhances cellular resistance to oxidative stress. Thus, this reduces damage to the body caused by oxidative stress. Wang et al. demonstrated that Nrf2 is upregulated in mesenchymal stem cells to protect against starvation-induced mitochondrial dysfunction and apoptosis, with Nrf2 possibly mediating its protective effects as a part of the anti-oxidative pathway [42]. Our results showed that in PDLSCs, the Nrf2-mediated anti-oxidative signaling pathway was activated to clear excess free radicals by upregulating NQO1, HO-1 and γ-GCS mRNA and protein.

In recent years, accumulating evidence has indicated that many drugs suppressed H_2_O_2_-induced oxidative stress and attenuated its induced cell injury via the expression of Nrf2 [43]. Research has also confirmed that Nrf2 itself influences the intrinsic and extrinsic apoptosis pathways at different levels [19]. Therefore, we believe that Nrf2 is extremely important in the process of cell apoptosis caused by oxidative stress. However, in our experiment, with an increasing concentration of H_2_O_2_, the Nrf2 antioxidant effect increased, as did the incidence of apoptosis. We suspect that the effect of Nrf2 on anti-apoptosis resistance was due to the factors related to the state of PDLSCs because apoptosis covered up the anti-oxidative effects and anti-apoptosis function of Nrf2. In this study, we executed Nrf2 overexpression. As a result, Nrf2 effectively increased the level of antioxidant molecules, such as NQO1, HO-1 and γ-GCS, and increased cell proliferation by inducing PCNA and Ki67. At the same time, it effectively restrained the intrinsic apoptosis pathway. Recently, a study demonstrated that Nrf2 overexpression/ARE activation could protect cells against apoptosis through anti-oxidation [44]. In contrast, after silencing the Nrf2 expression levels, we found that the PDLSC anti-oxidant capacity was significantly decreased, the cell proliferation levels were decreased and the intrinsic apoptosis pathway was upregulated. This agrees with the results of Kubben [45], who found that repression of the Nrf2-mediated anti-oxidative response was a key contributor to MSC apoptosis and a premature aging phenotype. This proves that Nrf2 in PDLSCs plays a core anti-oxidant role by being able to inhibit the intrinsic apoptotic pathway by inhibiting active caspase-9 and Bax. Therefore, inhibiting the apoptosis of PDLSCs plays a role in maintaining cell status and physiological functions.

## 4. Methods

### 4.1. Isolation and Culture of Human PDLSCs

Healthy premolars, for orthodontic reasons, were pulled and isolated at the Dental Clinic of School of Stomatology, Fourth Military Medical University, Xi’an, China. All subjects gave their informed consent for inclusion before they participated in the study. All procedures were performed according to institutional guidelines in accordance with the Declaration of Helsinki and were approved by the Ethics Committee of the Fourth Military Medical University, School of Stomatology (29 May 2015). An informed consent form that agreed to the contribution of their teeth for research purposes was signed by all donors, and the study was approved by the hospital’s ethics committee (license number: IRB-REV-2015038). After repeatedly rinsing the root surface with phosphate buffer saline (PBS), the middle third of the root was scraped to slice the periodontal membrane with a #11 sterile knife. It was washed with PBS three times and centrifuged at 800 rpm for 5 min, before the supernatant was removed. After enzymatic digestion for 20 min with 0.25% parenzyme, the tissues were incubated for 1 h with 1 mg/mL type I collagenase (Gibco, Grand Island, NY, USA) in alpha Minimum Essential Medium (α-MEM) (HyClone, Los Angeles, CA, USA) at 37 °C. The isolated cells were collected by brief centrifugation and were then resuspended in α-MEM supplemented with 10% FBS, 50 mg/mL streptomycin and 50 units/mL penicillin (HyClone). The culture medium was completely replaced every three days to replenish the non-adherent cells. Finally, the limiting dilution technique was used to purify stem cells from the primary cells. Cells at passages 3 (P3) were used in the subsequent investigations. 

### 4.2. Colony-Forming and Cell Viability Assay

Passage three PDLSCs were pre-incubated and transferred to culture dishes with a diameter of 10 cm (1000 cells per dish). Following this, the cells were cultured under normal conditions until day 14. On day 14, the cells in the dishes were fixed in 4% paraformaldehyde for 10 min and stained with 0.2% crystal violet (Sigma-Aldrich, Boston, MA, USA) for 15 h. The cell colonies were subsequently observed under a stereomicroscope (Olympus IX71, Olympus, Tokyo, Japan). Cell viability was analyzed using the 3-(4,5-dimethylthiazol-2-yl)-2,5-diphenyltetrazolium bromide (MTT) method. Briefly, the passage three PDLSCs were cultured in 96-well culture plates and the cells were stained with 5 mg/mL MTT (Sigma-Aldrich) at days 1, 3, 5, 7 and 9. The media were then carefully aspirated and 150 µl of dimethyl sulfoxide (DMSO) was added to solubilize the colored formazan product for 10 min. The optical density was read at 490 nm using a microplate reader (Floustar Optima; BMG Labtech, Ortenberg, Germany). The OS experimental cells were exposed to 125, 250, 500 and 1000 µM H_2_O_2_ for 2 h. After this, the cells were stained with 5 mg/mL MTT using the procedures described above.

### 4.3. Flow Cytometry

Passage three PDLSCs were suspended in 400 µL of PBS and incubated with each specific antibody. To evaluate surface markers, phycoerythrin (PE)-coupled antibodies against CD29, CD45, CD90 (12-0299, 12-9459 and 12-0909, respectively, eBioscience, Waltham, MA, USA), CD31, CD34, CD146 and STRO-1 (ab9498, ab81289, ab75769 and ab102969, respectively, Abcam, Cambridge, UK) were used. The secondary antibodies used included Cy3-conjugated goat anti-rabbit IgG and FITC-conjugated goat anti-mouse IgG (Zhongshan Co. Ltd, Beijing, China). The aliquots with corresponding dye-labeled isotype control antibodies served as the negative control. After incubation for 30 min at 4 °C in the dark, the cells were washed with PBS and then resuspended in 400 µL of PBS. Cell fluorescence was determined using a FACS-Aria flow cytometer (BD Biosciences, San Jose, CA, USA).

### 4.4. Detection of the Multi-Lineage Differentiation Ability of PDLSCs

Passage three PDLSCs were plated on 6-well plates. To determine their osteogenesis abilities, after 24 h, the cells were adhered, and the medium was switched to an osteoinductive differentiation medium (HUXMA-90031, Cyagen Biosciences, Waltham, MA, USA). The media were changed every two days. After osteogenic induction for 21 days, mineral nodules were detected by using alizarin red staining. To determine their adipogenesis abilities, passage three PDLSCs were adhered onto 6-well plates and the adipogenic induction medium (HUXMA-90021, Cyagen Biosciences) was used in place of the original culture medium. The medium was changed every two days. After 14 days, the lipid droplets were stained with Oil Red O solution.

### 4.5. Establishment of an Oxidative Stress Model and Determinations

Passage three PDLSCs were planted at a density of 105 in 6-well culture plates. After the cells were completely adherent, H_2_O_2_ was added to the media in the final concentrations of 125, 250, 500 and 1000 µmol/L to simulate the periodontitis environment as an in vitro model of oxidative stress. After culturing for 2 h, the medium with H_2_O_2_ was removed and the plates were rinsed with PBS three times for subsequent experiments. Non-H_2_O_2_ treated culture cells were utilized as a control group. ROS, SOD, MDA and GSH-Px levels were measured by fluorogenic assays to demonstrate the peroxidation levels.

### 4.6. Plasmids and Lentiviruses

The following plasmids and lentiviruses were designed and constructed by Genechem (Genechem Company, Shanghai, China): lentiviruses, Ubi-MCS-LV-NFE2L2-3FLAG-SV40-EGFP-IRES-puromycin for Nrf2 overexpression and Ubi-MCS-3FLAG-SV40-EGFP-IRES-puromycin for Nrf2 negative control (NC) overexpression; lentiviruses, hU6-MCS-LV-NFE2L2-RNAi-Ubiquitin-EGFP-IRES-puromycin (48515-1, 48516-1 and 48517-1) for Nrf2 silence and hU6-MCS-Ubiquitin-EGFP-IRES-puromycin for Nrf2 NC silence. The related sequence was amplified using the indicated primers: F:5′-GAGGATCCCCGGGTACCGGTCGCCACCATGATGGACTTGGAGCTGCC-3′ and R:5′-TCCTTGTAGTCCATACCGTTTTTCTTAACATCTGGCTTC-3′ for LV-NFE2L2 overexpression. For Nrf2 silence, shRNA (NFE2L2-RNAi #48515-1, 5′-TGACAGAAGTTGACAATTA-3′; NFE2L2-RNAi #48516-1, 5′-GAGAAAGAATTGCCTGTAA-3′; NFE2L2-RNAi #48517-1, 5′-GCAACAGGACATTGAGCAA-3′) targeting a specific region of human Nrf2 mRNA (NM_006164), and a scrambled negative control (sh-con077, 5′-TTCTCCGAACGTGTCACGT-3′) were cloned into vector. The overexpression and silence sequences were inserted into GV358 and GV248 plasmid vectors. In addition, we used blank GV358 and GV248 plasmid vectors as NC, respectively. Sequencing was performed to verify the correctness of the sequence. For lentivirus packaging, 293T cells were transferred with LV-NFE2L2 and LV-NFE2L2-RNAi plasmid vectors as well as the packaging plasmids, Helper 1.0 and Helper 2.0 (Genechem Company, Shanghai, China). The harvested lentiviruses were concentrated, purified and conserved at −80 °C.

PDLSCs that were grown to 50% confluence in a six-well plate were transfected with a lentivirus containing a Nrf2 overexpression or silence sequence in the presence of polybrene at a multiplicity of transfection of 40.

### 4.7. Fluorogenic Assay

Changes in intracellular ROS, MDA, SOD and GSH-Px levels were determined following the manufacturer’s protocol with the Reactive Oxygen Species Assay Kit (S0033, Beyotime, Shanghai, China), Lipid Peroxidation MDA Assay Kit (S0131, Beyotime), Total Superoxide Dismutase Assay Kit (S0101, Beyotime) and Cellular Glutathione Peroxidase Assay Kit (S0056, Beyotime). The active caspase-3, caspase-8 and caspase-9 units were detected with Caspase 3, 8 and 9 Activity Assay kits (C1116, C1152, C1158, Beyotime). To evaluate the caspase-3, caspase-8, and caspase-9 activities, isolated cells were homogenized in 100 µl of reaction buffer containing 2 mM Ac-DEVE-pNA, Ac-IETD-pNA and Ac-LEHD-pNA substrates, respectively, before being incubated at 37 °C for 2 h. Samples were measured with an ELISA reader at an absorbance of 530 nm for ROS, MDA and SOD, 412 nm for GSH-Px and at 405 nm for caspase-3, caspase-8 and caspase-9.

### 4.8. TUNEL Staining

Apoptotic PDLSCs were assessed by TUNEL analysis with an in-situ Cell Death Fluorescein Detection Kit (Roche, Basel, Swiss), following the manufacturer’s instructions. Briefly, after experimental stimulation, the cells were washed with PBS and fixed in 4% paraformaldehyde for 15 min. After rinsing with PBS, the cells were incubated with a reaction mixture solution for 30 min at 37 °C in the dark. Following this, they were mounted and analyzed under a confocal laser scanning fluorescence microscope (IX-71, Olympus, Tokyo, Japan). Positive and negative controls were treated with 0.1 mg/mL pancreatic DNase I (F. Hoffmann-La Roche Ltd., Diagnostics Division, Basel, Swiss) or labeling, respectively. The percentage of stain-positive cells was detected with an image analyzing system (Leica Qwin.Plus, Leica Microsystem Imaging, Cambridge, UK), and the percentage of TUNEL-positive cells was calculated from the number of total 2000 cells.

### 4.9. RNA Extraction, Reverse Transcriptase PCR and Real-Time PCR

Total RNA was extracted from cells using Tripure (Roche), according to the manufacturer’s instructions. Reverse-transcriptase (RT)-PCR was performed with 1 µg of total RNA, using oligo-deoxythymidine primers (Roche Diagnostics, Basel, Swiss) in 20 µL volumes at 37 °C for 20 min. Following this, gene expression was analyzed by quantitative real-time PCR (qRT-PCR) using SYBR^®^ *Premix Ex Taq^TM^* II (RR820A, Takara, Japan) in the CFX96 Real-time PCR machine (Bio-rad, Hercules, CA, USA). The qRT-PCR was conducted with GAPDH as the house-keeping gene and the mean values were derived using the formula 2^−ΔΔ*C*t^. The primer sequences are detailed in Table 1.

### 4.10. Western Blotting

Treated cells were washed with PBS and cytosolic protein extracts were prepared using Tripure (Roche) with a protease inhibitor cocktail and 1% sodium dodecyl sulfate (SDS). Protein concentrations were determined using the Bradford assay (Bio-Rad), according to the manufacturer's instructions. Protein lysate aliquots were separated on sodium dodecyl sulfate–10% polyacrylamide gels and Western blotting was performed. The proteins were transferred onto a 0.45 µm polyvinylidene difluoride (PVDF) membrane (Millipore, Boston, MA, USA) in transfer buffer (20 mm Tris, 150 mm glycine, 20% methanol, pH 8.0) at 4 °C and 100 V for 1 h. The membrane was blocked with 5% non-fat milk in Tris Buffered saline Tween (TBS-T) for 1 h at room temperature and incubated with primary antibodies overnight for rabbit anti-Nrf2 antibody (1:1000, 110 kDa, ab137550, Abcam), rabbit anti-HO-1 antibody (1:200, 32 kDa, sc-10789, Santa Cruz Biotechnology, Santa Cruz, CA, USA), rabbit anti-NQO-1 antibody (1:200, 30 kDa, sc-25591, Santa Cruz Biotechnology), rabbit anti-γ-GCS antibody (1:200, 73 kDa, sc-22755, Santa Cruz Biotechnology), mouse anti-Bcl-2 antibody (1:200, 26 kDa, sc-7382, Santa Cruz Biotechnology), mouse anti-Bax antibody (1:200, 23 kDa, sc-7480, Santa Cruz Biotechnology) and rabbit anti-Gapdh antibody (1:1000, 36kDa, ab181602, Abcam). After rinsing, the membrane was incubated with horseradish peroxidase (HRP)-conjugated IgG secondary antibodies (Zhuangzhi Company, Xi’an, China). Protein bands were detected using an enhanced chemiluminescence (ECL) system (Bio-Rad).

### 4.11. Statistical Analysis

Data are expressed as the mean values and standard deviations (SD). SPSS 22.0 (SPSS Inc., Chicago, IL, USA) was used for the statistical analysis and *p* values less than 0.05 were considered statistically significant for all statistical calculations. As per normal procedures, the data distribution was tested with the Shapiro-Wilk test at a 95% confidence level and Levene’s test was used to assess homogeneity of variance. One-way analysis of variance (ANOVA) was performed for multiple groups and Tukey’s post hoc test was applied for comparisons between groups. All experiments were repeated at least three times.

## 5. Conclusions

In conclusion, in the present study, we successfully simulated oxidative stress in PDLSCs and we identified that treatment with H_2_O_2_ led to apoptosis. Nrf2 alleviates PDLSCs through its anti-oxidative and anti-intrinsic apoptosis effects and by activating anti-oxidative enzymes and suppression of caspase-9, -3 and Bax. By increasing the levels of exogenous Nrf2 expression and strengthening its antioxidant ability, we can achieve success by realizing the optimization of seed cells. Thus, this improves periodontitis bone repair, which offers a new tissue engineering application strategy for PDLSCs.

## Figures and Tables

**Table 1:** Gene primers.

Genes	Forward Primer (5′–3′)	Reverse Primer (3′–5′)
*BSP*	GGCCACGATATTATCTTTACAAGCA	TCAGCCTCAGAGTCTTCATCTTCA
*OPG*	CTGCAGTACGTCAAGCAGGAGTG	TTTGCAAACTGTATTTCGCTCTGG
*ALP*	GGACCATTCCCACGTCTTCAC	CCTTGTAGCCAGGCCCATTG
*LPL*	CCAAACTGGTGGGACAGGATG	GCTCCAAGGCTGTATCCCAAGA
*PPAR-γ*	TGGAATTAGATGACAGCGACTTGG	TTGAATGTCTTCAATGGGCTTCAC
*PCNA*	GGCCGAAGATAACGCGGATAC	GGCATATACGTGCAAATTCACCA
*Ki67*	TGGGTCTGTTATTGATGAGCC	TGACTTCCTTCCATTCTGAAGAC
*Bax*	GCGAGTGTCTCAAGCGCATC	CCAGTTGAAGTTGCCGTCAGAA
*Bcl-2*	TCGCCCTGTGGATGACTGAG	CAGAGTCTTCAGAGACAGCCAGGA
*c-Fos*	TCTTACTACCACTCACCCGCAGAC	GGAATGAAGTTGGCACTGGAGAC
*Caspase3*	GACTCTGGAATATCCCTGGACAACA	AGGTTTGCTGCATCGACATCTG
*Caspase8*	CCAGAGACTCCAGGAAAAGAGA	GATAGAGCATGACCCTGTAGGC
*Caspase9*	TTCCCAGGTTTTGTCTCCTG	GGGACTGCAGGTCTTCAGAG
*Nrf2*	GATTCTGACTCCGGCATTTC	TCCCCAGAAGAATGTACTGG
*NQO1*	GGATTGGACCGAGCTGGAA	GAAACACCCAGCCGTCAGCTA
*HO-1*	TTGCCAGTGCCACCAAGTTC	TCAGCAGCTCCTGCAACTCC
*γ-GCS*	TTGCAGGAAGGCATTGATCA	GCATCATCCAGGTGTATTTTCTCTT
*GAPDH*	GCACCGTCAAGGCTGAGAAC	TGGTGAAGACGCCAGTGGA

## Supplementary Materials

Supplementary materials can be found at www.mdpi.com/1422-0067/18/5/1076/s1.
