# Supplementary material for: Nrf2 Inhibits Periodontal Ligament Stem Cell Apoptosis under Excessive Oxidative Stress
Source: Int J Mol Sci. 2017 May 17;18(5):1076. doi: 10.3390/ijms18051076 (PMC5454985; doi:10.3390/ijms18051076)
Supplement: Supplementary file 1 [file ijms-18-01076-s001.pdf]

Nrf2 Inhibits Periodontal Ligament Stem Cell Apoptosis under Excessive Oxidative Stress

Yanli Liu, Hongxu Yang, Yi Wen, Bingyi Li, Yinhua Zhao, Jing Xing, Min Zhang and Yongjin Chen

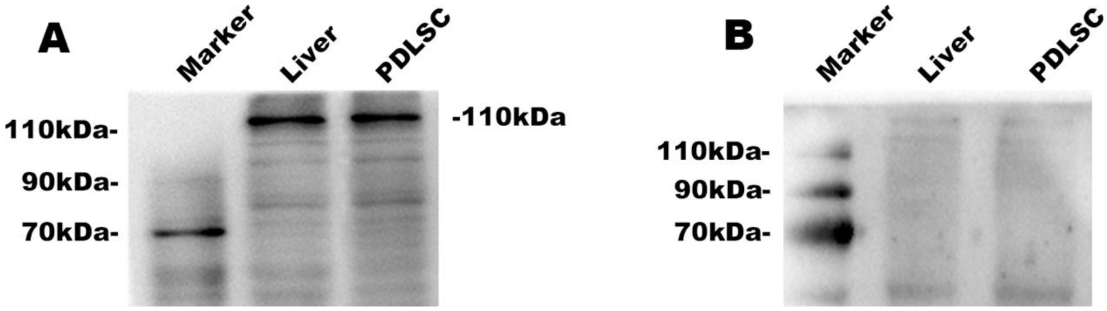

Figure S1. Nrf2 positive and negative control Western blot. (**A**) The positive control of Nrf2 expression assayed by western blot in liver tissue and PDLSCs; (**B**) The negative control of Nrf2 expression assayed by western blot without secondary antibody in liver tissue and PDLSCs.
